# Uric acid participating in female reproductive disorders: a review

**DOI:** 10.1186/s12958-021-00748-7

**Published:** 2021-04-27

**Authors:** Junhao Hu, Wenyi Xu, Haiyan Yang, Liangshan Mu

**Affiliations:** 1 Reproductive Medicine Center, The First Affiliated Hospital of Wenzhou Medical University, No.96 Fuxue Road, 325000 Wenzhou, People’s Republic of China; 2 School of Medicine, Zhejiang University, No.866 Yuhantang Road, 310058 Hangzhou, People’s Republic of China

**Keywords:** Uric acid, Polycystic ovary syndrome, Endometriosis, Pregnancy complications

## Abstract

Uric acid (UA) is the end metabolic product of purine metabolism. Early on, UA was considered to be a metabolite with a certain antioxidant capacity. As research has progressed, other properties of UA have been explored, and its association with many diseases has been found. The association between UA and kidney disease and cardiovascular disease is well established; however, there is still a paucity of reviews on the association between UA and the female reproductive system. An increasing number of epidemiological studies have shown elevated serum UA levels in patients with polycystic ovary syndrome (PCOS), endometriosis, etc. Additionally, serum UA can be used as a predictor of pregnancy complications and adverse foetal outcomes. An increasing number of animal experiments and clinical studies have revealed possible mechanisms related to the involvement of UA in certain female reproductive disorders: oxidative stress, chronic inflammation, mitochondrial dysfunction, etc. This article reviews the current mainstream mechanisms regarding the pathogenesis of UA and the role of UA in certain specific female reproductive disorders (direct involvement in the development of certain diseases or enhancement of other risk factors) in the hope of contributing to clinical prevention, diagnosis, treatment and improvement in prognosis.

## Background

Previous studies have revealed that physiological levels of uric acid (UA) have a protective function in vivo due to their antioxidant effect [1]. However, excessive serum UA levels are also a risk factor for kidney disease, cardiovascular disease, and metabolic syndrome [2, 3]. Some literature has reported that UA levels tend to be elevated to higher levels in some patients, such as those with polycystic ovary syndrome (PCOS), and correlate with clinical severity [4]. Additionally, trials targeting drugs to control UA levels have provided positive results, and some scholars have used UA levels to predict the occurrence of pregnancy complications and adverse maternal and infant risks [5–8]. These findings led us to question whether there is a close relationship between UA and female reproductive disorders. Based on these reports, we suggest that these reproductive disorders may be influenced by multiple factors, such as increased oxidative stress due to UA accumulation, inflammation, and mitochondrial dysfunction. In this paper, we review the evidence for the multiple adverse effects of UA levels on the female reproductive system and highlight the potential pathophysiological significance of UA in women.

### Uric Acid

**Fig. 1:**
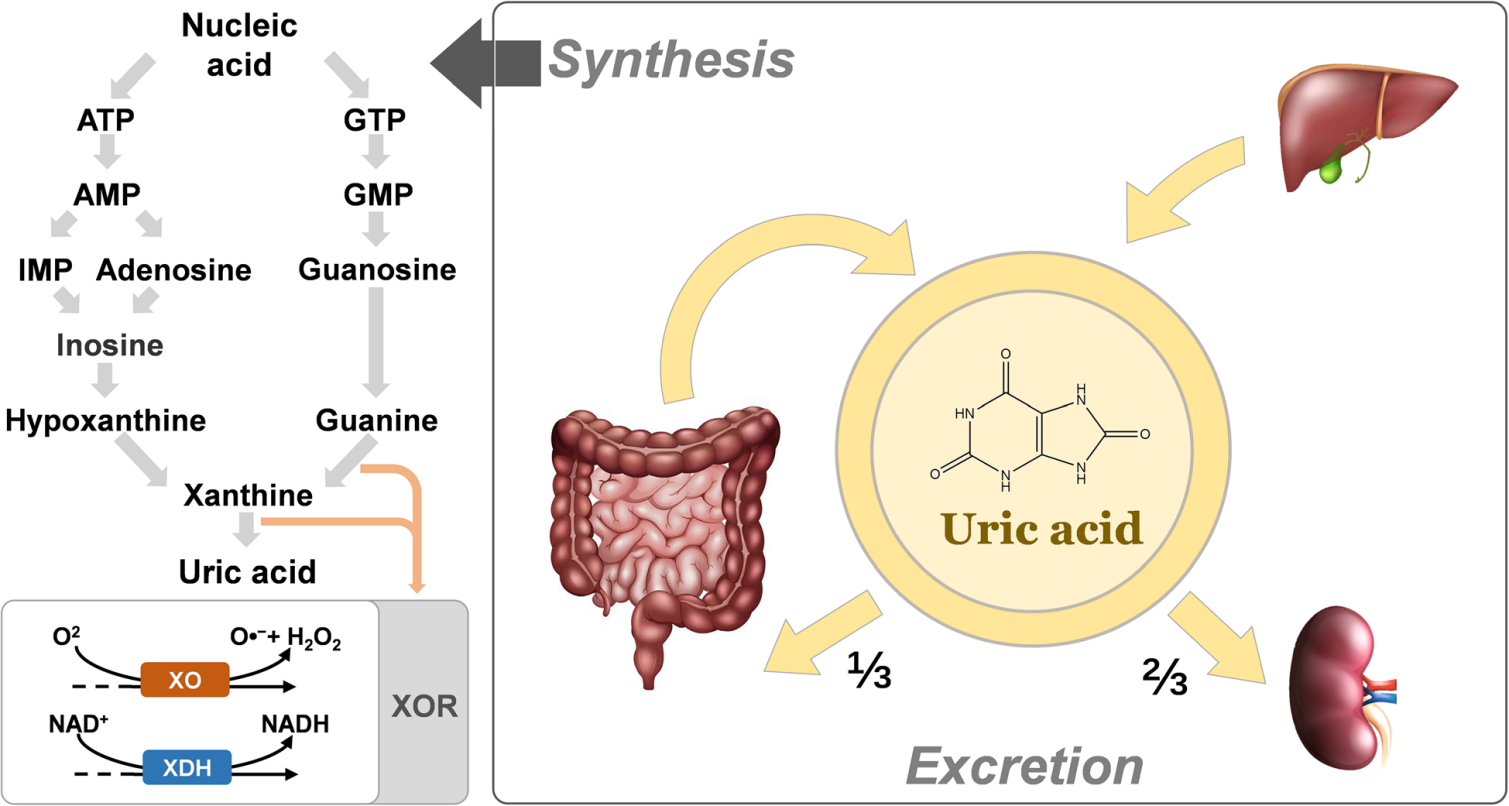
The metabolic process of uric acid

UA is a major product of purine metabolism catalysed by xanthine oxidoreductase (XOR). Plasma UA levels can be increased by exogenous pathways such as excessive intake of high-purine and high-fructose foods and are mainly affected by catabolism from endogenous pathways such as those in the liver and small intestine [9]. Hyperuricaemia is an elevated UA level in the blood, defined as wither > 7.0 mg/dL or > 6.0 mg/dL of serum UA for most studies. A variety of enzymes are involved in the synthesis of UA. The nucleotides in the cell are acted upon by nucleotidase to produce nucleosides, which are acted upon by nucleoside phosphorylase to produce bases. Among these purine bases, xanthine is produced by xanthine oxidase (XO), and UA is further produced by the action of this enzyme *(*Fig. 1*)*. XOR is the key enzyme in this process and has two convertible forms: xanthine dehydrogenase (XDH) and XO. XDH can convert NAD ^+^ to NADH via FADH_2_, while XO can convert O_2_ to hydrogen peroxide and free radical superoxide anion (O_2_^.^^−^) via FAD [10]. Thus, XOR can be one of the sources of reactive oxygen species (ROS) production. Moreover, XOR is active in the liver, intestine, and endothelium, so it can also cause oxidative stress and endothelial dysfunction. Approximately 2/3 of UA is excreted by the kidneys, and the rest is excreted by the gastrointestinal tract [11]. The main transporter associated with the reabsorption of UA in renal tubules is urate transporter 1 (URAT1), the main UA-secreting transporters are organic anion transporter 4 (OAT4) and glucose transporter 9 (GLUT9), and the amount of transporter can affect the level of serum UA to a certain extent [12].

UA is also found in the follicular fluid of the female ovary. Studies on buffalo ovaries have proven that disruption of the plasma-follicular barrier structure is associated with increased levels of UA [13]. In addition, hypoxanthine can inhibit oocyte meiosis, suggesting a possible decrease in fertility [14].

### Uric Acid in oxidative stress and mitochondrial dysfunction

UA is a known antioxidant and is a major contributor to antioxidant potential in vivo. UA not only removes singlet oxygen and free radicals but also reduces the damage of peroxynitrite to proteins involved in nearly half of the antioxidant effects in vivo [1]. UA has shown antioxidant effects in humans under many conditions and can rapidly return to its antioxidant status through the action of ascorbic acid [15]. At physiological concentrations, UA reduces the formation of oxyhaeme oxidants via peroxides that react with haemoglobin while preventing erythrocyte lipid peroxidation and protecting erythrocyte lysis due to peroxidation damage [10]. However, when the availability of antioxidants such as ascorbic acid decreases, UA will become an oxidant and participate in the occurrence of various pathological processes dominated by oxidative stress in the body.

Oxidative stress is related to the pathogenesis of many diseases, including cardiovascular disease, cancer, and neurodegenerative diseases [16, 17]. The presence of an oxidative stress state has also been reported in many female reproductive disorders, such as PCOS and pre-eclampsia (PE) [18, 19]. Oxidative stress occurs mainly from an imbalance between ROS production and the antioxidant defence system, and UA also seems to be involved in this process. As previously mentioned, UA is produced by XO and XDH, which together catalyse the oxidation of purines. Under normal conditions, XDH is the main substance involved in this reaction; however, the activity of XO is enhanced under specific conditions, such as oxidative stress. XO is an important source of superoxide radicals. It can utilize molecular oxygen to produce UA along with active superoxide radicals and hydrogen peroxide [11]. Generated ROS can not only alter the normal function of mitochondria but also in turn produce excess ROS that may diffuse into the cytoplasm [20]. When the production of these superoxide free radicals increases excessively or is absolutely or relatively insufficient, the circulating ROS will increase significantly, leading to the development of oxidative stress in the body.

In addition to inducing oxidative stress, high concentrations of serum UA also affect lipid synthesis and lipid oxidation distortion. High levels of UA stimulate the NADPH oxidase isoform NADPH oxidase 4 (NOX4) in mitochondria, which increases superoxide production and consequently decreases aconitase activity. This leads to the accumulation of its substrate citric acid, promoting its re-catabolism to acetyl-CoA for lipid synthesis [21]. Excessive fat deposition promotes the production of pro-inflammatory factors such as IL-1β, which can further mediate lipid oxidative stress through the NF-κB and NADPH oxidase pathways [22]. In addition, disorders of lipid metabolism are associated with an imbalanced state of AMP-activated kinase (AMPK) and AMP deaminase (AMPD), a pair of enzymes with mutually antagonistic effects. Intracellular UA accumulation reverses physiological homeostasis and promotes fat accumulation and gluconeogenesis by activating AMPD and inhibiting AMPK [23]. Whereas adipose tissue is rich in XOR, obesity increases XOR mRNA expression, which in turn promotes UA secretion [21].

Abnormal lipid metabolism, on the one hand, produces lipotoxicity, which has a direct adverse effect on cells, and on the other hand can produce large amounts of ROS, which induces stress damage to intracellular organelles leading to cell death [1, 22]. Nakagawa et al. [24] reduced the production of UA by the XO inhibitor allopurinol while simultaneously inhibiting the progression of hypertriglyceridaemia.

### Uric Acid in the development of inflammation

High levels of UA can lead to systemic sterile inflammation and may also become a pro-inflammatory factor. The mainstream view is that high levels of UA form monosodium urate (MSU) around cells through extracellular sodium reactions. Apoptosis-associated speck-like protein containing a caspase-associated recruitment domain (ASC) recruits caspase-1 and, together with NLR family pyrin domain containing 3 (NLRP3), forms NLRP3 inflammatory vesicles that convert MSU-produced pro-IL-1 into IL-1β, which triggers the inflammatory response [25]. However, the distribution of NLRP3 in cells is relatively restricted. Its expression in the female reproductive system is found only in non-keratinized epithelial cells of the uterine cervix [26]. In addition, it has been reported that pregnant women with concurrent PE often have high levels of MSU, which can trigger placental inflammation through NLRP3 inflammatory vesicles and produce factors such as IL-1β to induce apoptosis in trophoblast cells [27].

In addition to promoting inflammation in the form of MSU crystals, soluble UA in adipocytes can also mediate lipid oxidation by activating mitogenic protein kinases such as p38 mitogen-activated protein kinase (p38 MAPK) and extracellular signal-regulated kinase 1/2 (ERK1/2) through NADPH oxidase activation [28]. In other cells, this signalling pathway can also induce the production of various chemokines and inflammatory markers, including monocyte chemotactic protein 1 (MCP-1), C-reactive protein (CRP), IL-1, IL-6, IL-18, and tumour necrosis factor-α (TNF-α), which trigger the disruption of lipid metabolism [29, 30].

**Fig. 2:**
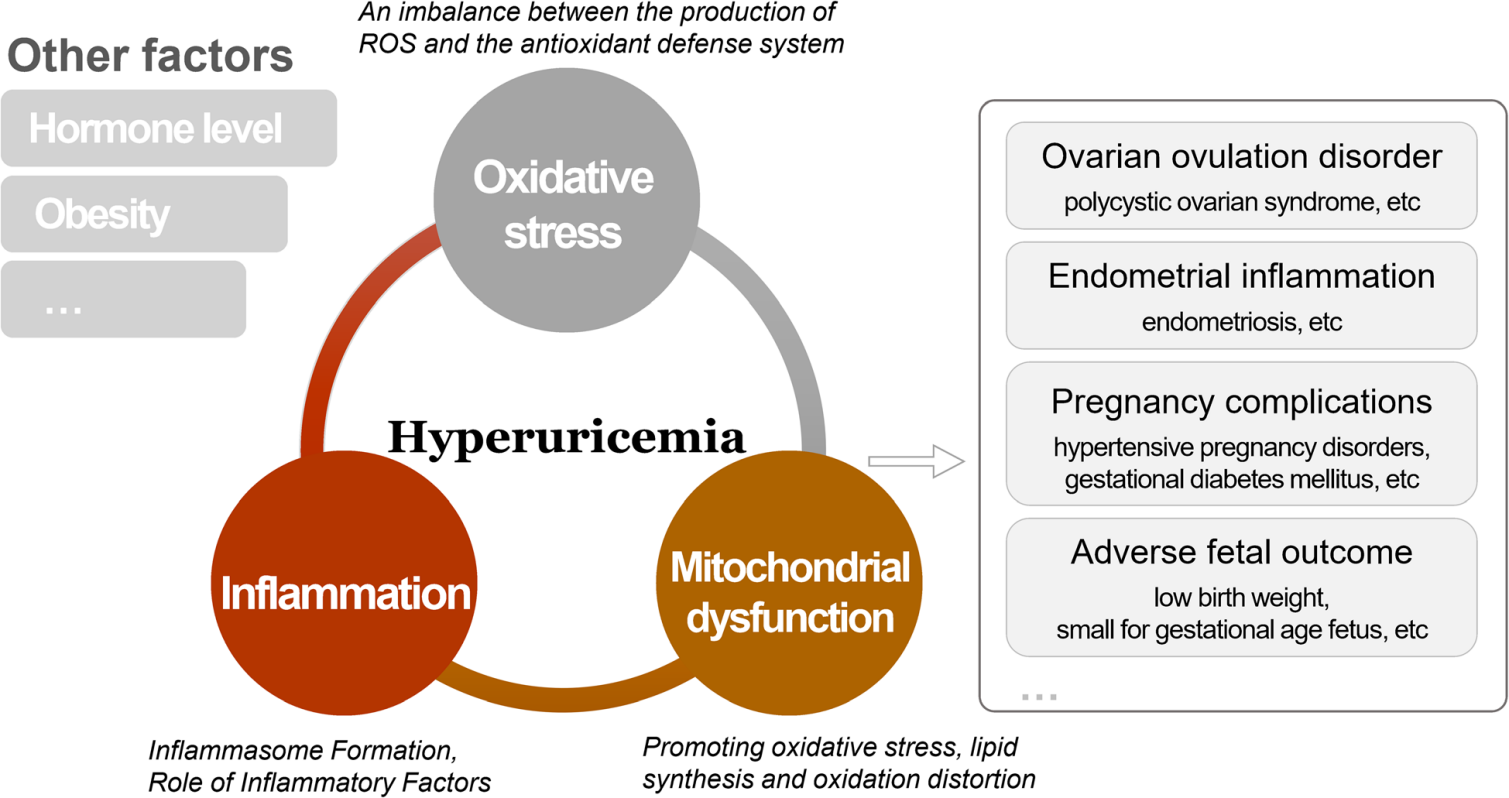
Hyperuricemia is linked to female reproductive health, mainly through oxidative stress, inflammation and mitochondrial dysfunction

UA serves as an end product of purine metabolism reflecting the metabolic state of the body, and it can maintain the level of oxidation in the body under physiological conditions. However, excessive accumulation of UA can cause damage to multiple systems throughout the body. In reproductive system diseases, although these mechanisms are still unclear, we currently believe that UA may be involved in key aspects of oxidative stress, inflammation, and metabolic disorders in vivo, leading to the development of various pathological conditions *(*Fig. 2*)*.

### Hormone level effects on Uric Acid levels

Reproductive hormones also affect serum UA levels. Although there is considerable disagreement on the issue of specific hormones and UA levels, the prevailing view is that high levels of testosterone are strongly associated with elevated serum UA levels and hyperuricaemia. Testosterone may induce functional alterations in the renal UA reabsorption system through increased expression of URAT1, sodium-coupled monocarboxylate transporter 1, and/or 2  (SMCT1 and/or 2) or GLUT9, among others [31]. Animal studies also suggest that testosterone may increase serum UA levels by inducing the hepatic metabolism of purine nucleotides [32]. High UA levels and hyperuricaemia are common in PCOS patients and are usually accompanied by hyperandrogenaemia [4]. Excessive androgen production by the ovaries and adrenal glands is an important cause of a large amount of follicle atresia, which ultimately leads to ovulation disorders in women with PCOS [33]. In addition to the conventional treatment of UA, the use of anti-androgenic contraceptives reduces serum UA levels in obese PCOS patients and significantly reduces the clinical severity [8]. The effect of serum UA on testosterone levels is still unknown.

The link between oestradiol and UA is controversial. High UA levels are observed in menopausal women under normal conditions and may also be associated with postmenopausal insulin resistance (IR), obesity (BMI ≥ 25 kg/m^2^), and ethanol intake [34]. Hormone replacement therapy with oestrogen reduces serum UA levels in postmenopausal patients with hyperuricaemia [35]. These pieces of evidence suggest a potential effect of oestrogen. Oestrogen seems to increase the excretion of UA from the kidneys and intestines, and at the same time, it reduces the production of UA by inhibiting the XO system during hypoxia and stabilizing lipid metabolism [36–38]. However, it has also been suggested that the high prevalence of hyperuricaemia in older women is only associated with ageing and not with menopause [39]. Some studies have similarly shown opposing results [4, 40]. Larger samples are still needed for corroboration.

The relationship between follicle-stimulating hormone, luteinizing hormone and UA levels is also controversial. Few studies have reported a positive relationship between increased levels of follicle-stimulating hormone and luteinizing hormone and UA accumulation [4, 40]. Notably, plasma sex hormone-binding globulin (SHBG) is significantly lower in postmenopausal patients with elevated serum UA levels [41]. Changes in hormones in patients with hyperuricaemia may also be associated with changes in their plasma SHBG levels. SHBG can also bind free androgens to reduce their levels and control their bioavailability to indirectly affect the UA concentration. Fujihara et al. [42] suggested that a lower concentration of SHBG (46.5 nmol/L) was a predictor of hyperuricaemia, which may lead to reduced production of SHBG via induced inactivation of AMPK in the liver [43].

### Uric acid in polycystic ovarian syndrome (PCOS)

Serum UA accumulation is closely related to ovarian ovulation disorders. PCOS is a common metabolic disease characterized by hyperandrogenism, oligo-ovulation, and polycystic ovarian morphology, often accompanied by an adverse clinical risk of miscarriage and many pregnancy complications [44]. Frequent abnormal lipid metabolism and abnormal glucose metabolism that accompany PCOS also increase the risk of cardiovascular disease, diabetes, and other chronic diseases [45]. Hyperuricaemia is one of the important characteristics observed in these patients. Several studies have shown that serum UA levels are significantly higher in PCOS patients than in non-PCOS women of normal reproductive age, especially in those patients with obesity, which is believed to be an important factor affecting UA concentrations in PCOS patients [4, 46]. Liu et al. [47] summarized the role of UA in the development of PCOS in patients, emphasizing its potential mechanisms in oxidative stress, inflammatory promotion, endothelial injury, and thrombosis in cardiovascular disease progression in these patients.

High serum UA levels in PCOS patients are closely related to androgen excess and IR. Androgens may increase serum UA levels by inducing hepatic metabolism of purine nucleotides and enhancing purine renewal in the kidney [32, 48]. The use of anti-androgenic contraceptives significantly reduces serum UA levels in these patients [8]. Hyperuricaemia is also an important marker of IR. Serum UA levels are negatively associated with insulin sensitivity [49, 50]. PCOS patients with combined IR tend to have higher levels of insulin in the blood. High insulin levels not only reduce the renal excretion of UA but also increase the potential for androgen production [51, 52].

IR is prevalent in women with PCOS independent of obesity, which can also be observed in many patients with hyperuricaemia [53]. Possible mechanisms by which hyperuricaemia may cause or aggravate IR are as follows: (i) high UA levels reduce tissue phosphorylated protein kinase B (pAkt) levels, causing impairment of the protein kinase B/endothelial nitric oxide synthase (Akt/eNOS) signalling pathway and resulting in decreased nitric oxide (NO) production, which decreases blood flow to skeletal muscle and reduces its uptake of glucose [54]. Animal experiments have also found that NO induces IR and leads to impaired vasodilation in hyperuricaemic rats [55]. (ii) UA can form NLRP3 inflammatory vesicles and release various pro-inflammatory factors, which can impair insulin signalling. Animal experiments revealed that disruption of the NLRP3 gene in obese mice resulted in inactivation of the insulin phosphoinositide 3 kinase/protein kinase B (PI3K/Akt) pathway and ERK1/2, resulting in decreased cellular sensitivity to insulin [56, 57]. Moreover, the activation of ERK1/2 downregulates the expression of peroxisome proliferator-activated receptor α in rat liver, which reduces fatty acid β-oxidation and triggers impaired fatty acid oxidation and triglyceride accumulation, ultimately causing obesity and hypertriglyceridaemia [58]. Obesity is an important factor affecting UA levels in PCOS; at the same time, elevated UA increases the risk of developing hypertriglyceridaemia [59]. (iii) In addition, certain effects of high levels of fatty acids in plasma can impair insulin secretion due to pancreatic β-cell dysfunction [60]. (iv) Finally, high levels of UA also reduce adiponectin levels and increase the risk of type 2 diabetes [61].

Hyperuricaemia in patients with PCOS was also observed to be accompanied by significant levels of oxidative stress, especially in the presence of obesity [46, 49]. These patients tend to have significant elevations in oxidative stress biomarkers, including NO, malondialdehyde, and many adaptive changes in antioxidative stress markers [62]. Malondialdehyde is a stable end product of lipid peroxidation, suggesting an imbalance in lipid oxidation. In this way, the unbalanced oxidation level caused by the accumulation of UA exacerbates the occurrence of inflammation. In the inflammatory state of PCOS, monocytes release more ROS due to hyperglycaemia, which then reactivates cytokines such as TNF-α and inflammatory transcription factors. Finally, in this vicious cycle, UA mediates the development of IR and hyperandrogenaemia [51].

### The potential link between Uric Acid, IL-1β, and endometriosis

**Fig. 3:**
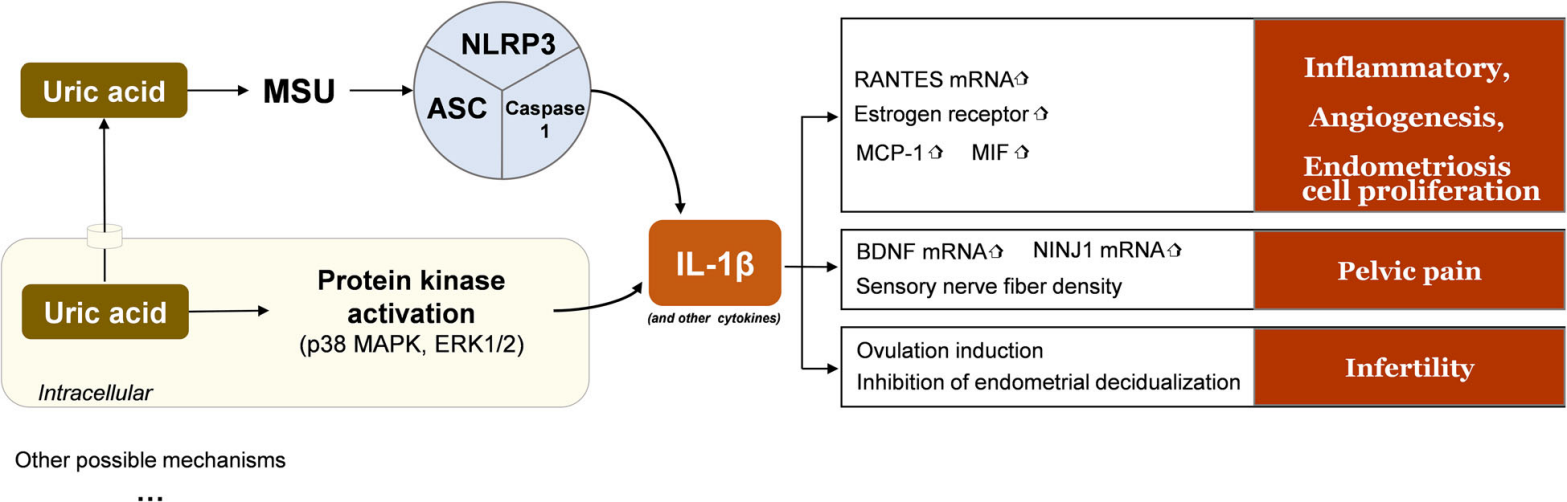
The potential mechanisms of uric acid in the pathogenesis of endometriosis

Endometriosis is considered to be an inflammatory, immune, and haemorrhagic disease with the main clinical manifestations of dysmenorrhea, chronic pelvic pain, menstrual abnormalities, and infertility. Accumulating evidence suggests that IL-1β plays an important role in the development of endometriosis *(*Fig. 3*)*. IL-1β is produced when UA forms inflammasomes (mentioned above). Serum IL-1β levels were significantly higher in women with deep infiltrating endometriosis than in normal women in the control group [63]. IL-1β may induce thymic stromal lymphopoietin secretion by promoting regulation upon activation of normal T cell expression and secreted factor mRNA expression, allowing monocytes and T cells to accumulate and activate Th2 immune responses, producing a local inflammatory response [64, 65]. Akoum et al. [66] reported that oestrogen can synergize with IL-1β to enhance local inflammatory responses. On the one hand, the number of oestrogen receptors on macrophages in the peritoneal fluid is associated with the production of IL-1β, and on the other hand, oestrogen promotes the secretion of MCP-1, which enhances the recruitment of macrophages [67]. In addition to pro-inflammatory effects, IL-1β plays a role in promoting angiogenesis and the proliferation of endometrial cells [68]. This may be related to the promotion of macrophage migration inhibitory factor synthesis by IL-1β.

Women with endometriosis often have prolonged, severe pelvic pain that is not relieved by analgesic drugs. This may be because IL-1β increases brain-derived neurotrophic factor (BDNF) and the nerve injury-inducible protein 1 (Ninj1) through a signal transduction pathway mediated by c-Jun N-terminal kinase (JNK) and NF-κB, which in turn stimulates the associated nerves and causes pelvic pain [69, 70]. It has also been found that IL-1β increases the density of sensory nerve fibres and decreases the density of sympathetic nerve fibres [71]. In mice with endometriosis, the lesions were reduced in size and pain was relieved with significantly lower IL-1β levels following the use of NF-κB activation inhibitors such as norethindrone [72].

Up to approximately one-half of women with endometriosis have infertility [73]. It has been suggested that the imbalanced state of IL-1β and its decoy inhibitory receptors may contribute to infertility in patients with endometriosis. Higher concentrations of IL-1β and lower concentrations of IL-1 receptor 2 (decoy inhibitory receptor type 2 of IL-1) were observed in the peritoneal fluid of patients with endometriosis with infertility compared to normal women [74]. In addition, IL-1β at certain levels induces ovulation and inhibits endometrial metaplasia, which may be detrimental to embryo implantation and pregnancy maintenance in normal women and women with endometriosis, ultimately leading to infertility [75].

### Uric Acid and Pregnancy complications

In the early stages of normal pregnancy, serum UA levels decrease due to increased plasma volume, increased renal clearance, and the urate excretion promoting effect of oestrogen. Serum UA stabilizes at a certain level during most of the second trimester. In late gestation, serum UA increases rapidly due to the excretion of foetal metabolites through the mother [76]. Almost half of women with hypertensive disorders of pregnancy (HDPs) or gestational diabetes mellitus (GDM) have significantly increased UA concentrations in pregnancy [6, 7].

HDPs include four conditions: gestational hypertension, PE, chronic hypertension and chronic hypertension with superimposed PE. PE is a severe HDP that begins with impaired maternal uteroplacental vascular formation. If not treated early, it may progress to eclampsia as well as maternal and foetal in utero growth restriction and death. Many pregnant women with PE commonly have elevated UA levels or even hyperuricaemia. There are multiple potential sources of increased UA in PE, the main ones are as follows [77, 78]: (i) Overproduction of vasoconstrictor substances leads to decreased renal UA secretion, and decreased renal blood flow and glomerular filtration decrease UA clearance. (ii) Poor trophoblast invasiveness. Hypoxia stimulates massive production of lactic acid, leading to acidosis that can impede UA secretion. (iii) Apoptotic homeostasis occurs when trophoblast cells are stimulated by hypoxia and oxidative stress. UA, a major product of purine metabolism, will be produced in increased amounts. (iv) Under these conditions, increased XO/XDH activity, which is involved in purine metabolism, directly increases the production of UA.

Similarly, in PE patients, high UA levels affect the normal function of blood vessels and the placenta mainly through oxidative stress and inflammation. UA induces oxidative stress, which is considered to be a key link in PE. On the one hand, oxidative products promote the production of vasoconstrictive substances, activating the local renin-angiotensin system in human endothelial vascular cells including angiotensin II. On the other hand, it leads to decreased production and bioavailability of diastolic substances (e.g., NO, prostacyclin) inducing endothelial dysfunction [79]. Doughan et al. [20]. proposed a model of angiotensin II-induced mitochondrial dysfunction. Angiotensin II activates NADPH oxidase through protein kinase C (PKC)-dependent activation, leading to ROS production and mitochondrial dysfunction, which in turn further activates NADPH oxidase, resulting in increased cellular oxidation levels and decreased NO bioavailability. Among these, the effect of UA on NO production not only affects the maternal vascular system but is also crucial for changes in trophoblast cells during placental development, which may be involved in the development of HDPs and adverse foetal outcomes [30]. In hyperuricaemic rats, UA reduces endothelial NO synthase activity to limit NO availability and upregulates cyclooxygenase-2 (COX-2) expression by increasing the production of the vasoconstrictor thromboxane, impairing placental perfusion and inhibiting foetal growth [80].

High UA levels also have a profound effect on endothelial cell migration, proliferation, and apoptosis. UA significantly inhibited serum-induced proliferation in human umbilical vein endothelial cells and their migration [30]. At the same time, proliferation and apoptosis of vascular smooth muscle cells in an inflammatory state cause vascular remodelling and ultimately widespread endothelial cell dysfunction [29]. UA-induced inflammation may be widely relevant. In addition to the observation of increased multiple inflammatory markers, Stodle et al. [81] also reported enhanced NLRP3 inflammatory vesicle activation caused by UA crystals in the trophoblastic layer of the placental syncytium in PE, highlighting an important link to inflammation.

GDM is a type of diabetes that is not present before pregnancy and occurs during pregnancy. Although it may disappear after delivery, women who have had GDM are at an increased risk of developing type 2 diabetes later. Early pregnancy UA levels can be used to some extent as a secondary predictor of GDM and may be partially involved in the development of GDM [82]. High UA levels that persist after delivery are also a high-risk factor for type 2 diabetes mellitus. It was found that patients with GDM have active purine metabolism and higher levels of UA in serum and amniotic fluid [83, 84]. The latter was also positively associated with BMI and glucose levels [85]. Possible mechanisms by which high serum UA levels lead to glucose metabolism disorders and IR include mitochondrial dysfunction, lipid oxidative stress, inflammatory mediator production such as TNF-α, and reduced lipocalin levels [86–88].

### Uric Acid and adverse foetal outcomes

Serum UA in some cases can directly affect foetal growth. In addition to oxidative stress and inflammation affecting the vasculature and placenta, as mentioned earlier, other causes include the following: **(i)** The direct toxic effects of UA on foetal growth. **(ii)** Elevated UA levels can affect the transport of nutrients. For example, UA-induced oxidative stress can inhibit mammalian target of rapamycin (mTOR) signalling and reduce amino acid transport to the foetus [89]. **(iii)** Finally, elevated UA levels can also affect hormone levels in foetuses, especially growth hormones [90].

Serum UA levels have also been associated with the risk of preterm birth in pregnant women. In a case-control study by Roberts et al. [91], hyperuricaemia was found to increase the risk of preterm delivery and low birth weight of the infant. Pregnant women with PE who had low to moderate levels of UA concentrations at admission had an increased likelihood of prolonged pregnancy [92]. Lower foetal birth weight may also be associated with preterm delivery, where the shortened gestation time allows the foetus to be born before it is fully developed in utero.

The relationship between UA and inflammation has already been mentioned above, including the involvement of multiple inflammatory factors and the formation of inflammatory vesicles. Several studies have found that elevated IL-1β levels may increase the risk of preterm birth. The evidence that IL-1β increases the risk of preterm birth is as follows: (i) IL-1β expression is increased in the amnion, chorionic villus, cervix, and myometrium after normal delivery [93]. IL-1β can induce uterine smooth muscle contraction by increasing COX-2 expression and promoting prostaglandin synthesis [80]. (ii) IL-1β can enhance local progesterone metabolism in some cells by regulating 20α-hydroxysteroid dehydrogenase activity, which decreases local progesterone levels to activate uterine muscle [94]. (iii) Animal studies have demonstrated that IL-1β alone can induce preterm labour, while the use of IL-1β receptor antagonists can prevent this effect [95]. (iv) IL-1 receptor antagonist has a linear relationship with IL-1β, while studies have found a high predictive value of cervicovaginal fluid and blood IL-1 receptor antagonist levels for preterm birth risk [96, 97]. Thus, we speculate that elevated UA levels may indirectly affect the course of pregnancy and increase the risk of preterm delivery.

Elevated serum UA levels are considered to be a predictor of maternal and infant complications, so early measurement of serum UA may be beneficial in the diagnostic treatment of this clinical syndrome. The sensitivity from the study by Bellos et al. [5] for predicting adverse perinatal outcomes in patients with PE with high UA levels ranged from 67.3 to 82.7 %, and the specificity ranged from 47.7 to 70.7 %. In women with PE/eclampsia, high UA levels are considered in most studies to be a good predictor of foetal/neonatal complications. A cohort study by Le et al. [98] found that serum UA levels at a threshold of 393 mmol/l may be predictive of adverse outcomes such as preterm birth, low Apgar scores, intrauterine growth restriction, and neonatal death in women with PE.

Amniotic fluid UA monitoring is also considered to be of value in predicting foetal growth. In early gestation, amniotic fluid is mainly derived from the plasma component of the embryo. As the organs of the embryo begin to mature and develop, the metabolites of the foetus gradually increase, as do the levels of amniotic fluid UA. It has been suggested that amniotic fluid UA is an important predictor of infant birth weight in mid-gestation [99]. In addition, amniotic fluid UA also reflects maternal pre-pregnancy BMI, which correlates with maternal nutritional status and oxidative stress levels [100]. In late gestation, the UA concentration in maternal amniotic fluid is significantly higher than that in maternal venous serum or cord serum, which may be determined mainly by the urine produced by foetal excretion. The amniotic fluid UA concentration may also be an indicator of foetal maturity due to its ability to reflect the maturity of the kidney. Harrison [101] used amniotic fluid UA to detect up to 79 % of mature foetuses. Animal studies by Koski et al. [102] confirmed the relationship between elevated amniotic fluid UA levels and foetal weight, emphasizing that the amniotic fluid composition may be predictive of foetal growth and metabolic maturity.

## Conclusion and future perspectives

In this review, we tried to summarize the relationship between UA and female reproductive diseases, focusing on some of the characteristics of UA and its possible mechanisms in the development of PCOS, endometriosis, pregnancy complications, and other diseases. We believe that there is a close interaction between abnormal UA levels in patients with these diseases and various symptoms and long-term complications. We suspect that there is a certain correlation between UA levels and oxidative stress, inflammation, mitochondrial dysfunction, and so on. These factors influence and promote each other, forming a vicious circle. We hope our results can be helpful in showing possibilities for potential prevention, treatment, and prognostic assessment.

Our understanding of the role of UA in the reproductive system is also growing as more literature is added. Serum UA levels have been proposed to predict the occurrence of PE and adverse maternal and foetal outcomes [5]. A model containing UA and hypoxanthine has also been constructed to diagnose the prognosis of minimal/mild endometriosis with a sensitivity of 66.7 % and specificity of 90.0 % [103]. In some trials targeting low seminal plasma UA levels in infertile male patients, antioxidant protocols have been used with overall positive results. Anti-androgen drugs in PCOS patients also significantly alleviated the state of high UA levels. Nevertheless, more well-designed trials are needed to assess the potential of these regimens.

## Data Availability

Not applicable.

## Abbreviations

UA: Uric Acid
PCOS: Polycystic Ovary Syndrome
XOR: Xanthine Oxidoreductase
XO: Xanthine Oxidase
XDH: Xanthine Dehydrogenase
ROS: Reactive Oxygen Species
URAT1: Urate Transporter 1
OAT4: Organic Anion Transporter 4
GLUT9: Glucose Transporter 9
PE: Pre-eclampsia
NOX4: NADPH Oxidase 4
AMPK: AMP-activated Kinase
AMPD: AMP Deaminase
MSU: Monosodium Urate
NLRP3: NLR Family Pyrin Domain Containing 3
p38 MAPK: P38 Mitogen-Activated Protein Kinase
ERK1/2: Extracellular Signal-Regulated Kinase 1/2
MCP-1: Monocyte Chemotactic Protein 1
CRP: C-Reactive Protein
TNF-α: Tumor Necrosis Factor α
SMCT1 and/or 2: Sodium-Coupled Monocarboxylate Transporter 1 and/or 2
IR: Insulin Resistance
SHBG: Sex Hormone-binding Globulin
pAkt: Phosphorylated Protein Kinase B
Akt: Protein Kinase B
eNOS: Endothelial Nitric Oxide Synthase
NO: Nitric Oxide
PI3K: Phosphoinositide 3 Kinase
HDPs: Hypertensive Disorders of Pregnancy
GDM: Gestational Diabetes Mellitus
BDNF: Brain-Derived Neurotrophic Factor
Ninj1: Nerve Injury-Inducible Protein 1
JNK: C-Jun N-Terminal Kinase
PKC: Protein kinase C
COX-2: Cyclooxygenase-2
mTOR: Mammalian Target of Rapamycin
